# Comparison of clinical outcomes and safety between laminectomy with instrumented fusion versus laminoplasty for the treatment of multilevel cervical spondylotic myelopathy

**DOI:** 10.1097/MD.0000000000014651

**Published:** 2019-02-22

**Authors:** Xiang Lin, Jie Cai, Chuan Qin, Qinghua Yang, Zengming Xiao

**Affiliations:** a Department of Musculoskeletal Oncology, Affiliated Tumor Hospital of Guangxi Medical University; b Department of Spine Surgery, The First Affiliated Hospital of Guangxi Medical University, Nanning, China.

**Keywords:** fusion, laminectomy, laminoplasty, meta-analysis, multilevel cervical spondylotic myelopathy

## Abstract

**Study design:**

Systematic review and meta-analysis.

**Objectives:**

Posterior laminectomy with instrumented fusion and laminoplasty are widely used for the treatment of multilevel cervical spondylotic myelopathy (MCSM). There is great controversy over the preferred surgical method. The purpose of this study is to evaluate the clinical outcomes and safety between laminectomy with instrumented fusion and laminoplasty for the treatment of MCSM.

**Methods:**

Related studies that compared the effectiveness of laminectomy with instrumented fusion and laminoplasty for the treatment of MCSM were acquired by a comprehensive search in PubMed, Embase, the Cochrane library, CNKI, VIP, and WANFANG up to April 2018. Included studies were evaluated according to eligibility criteria. The main endpoints included: preoperative and postoperative Japanese Orthopedic Association (JOA) scores, preoperative and postoperative visual analog scale (VAS), preoperative and postoperative cervical range of motion (ROM), preoperative and postoperative cervical curvature index (CCI), overall complication rate, C5 nerve palsy rate, axial symptoms rate, operation time and blood loss.

**Results:**

A total of 15 studies were included in this meta-analysis. All of the selected studies were of high quality as indicated by the Newcastle–Ottawa scale (NOS). Among 1131 patients, 555 underwent laminectomy with instrumented fusion and 576 underwent laminoplasty. The results of this meta-analysis indicated no significant difference in preoperative and postoperative JOA scores, preoperative and postoperative VAS, preoperative and postoperative CCI, preoperative ROM and axial symptoms rate. However, compared with laminoplasty, laminectomy with instrumented fusion exhibited a higher overall complication rate [RR = 1.99, 95% confidence intervals (CI) (1.24, 3.21), *P* <.05], a higher C5 palsy rate [RR = 2.22, 95% CI (1.30, 3.80), *P* <.05], a decreased postoperative ROM [SMD = −1.51, 95% CI (−2.14, −0.88), *P* <.05], a longer operation time [SMD = 0.51, 95% CI (0.12, 0.90), *P* <.05] and increased blood loss [SMD = 0.47, 95% CI (0.30, 0.65), *P* <.05].

**Conclusion:**

These results suggested that both posterior laminectomy with instrumented fusion and laminoplasty were determined to be effective for MCSM. However, laminoplasty appeared to allow for a greater ROM, lower overall complication and C5 palsy rates, shorter operation time and lower blood loss. Future well-designed, randomized controlled trials are still needed to further confirm our results.

## 1 Introduction

Multiple level (≥3 segments) cervical spondylotic myelopathy (MCSM) usually leads to the gradual deterioration of spinal cord function.^[1]^ Early intervention with surgery can improve the prognosis of MCSM patients.^[2]^ In treating MCSM, an anterior approach results in a more complicated surgical performance, associated with increased complications such as internal graft dislocation and dysphagia. This finding explains why the posterior approach is more commonly used by surgeons.^[3]^

For the posterior approach, laminectomy alone is the standard therapy for treating MCSM. To reduce the rate of postoperative cervical kyphosis and segmental instability, instrumented fusion such as lateral mass screw fixation is performed following laminectomy.^[4]^ Laminoplasty is considered an alternative method using a posterior approach for treating MCSM, providing a greater postoperative cervical range of motion (ROM) and decreased destruction of the posterior structures of the cervical spine.^[5]^ Both procedures are widely applied for treating MCSM because of the satisfactory clinical outcomes over time.

Some studies indicate that laminoplasty is superior to laminectomy with instrumented fusion, but other studies reveal different conclusions. The primary goal of this meta-analysis is to pool the most recently published studies in order to determine whether laminectomy with instrumented fusion or laminoplasty is significantly better in terms of clinical and radiographic outcomes and complications in treating patients with MCSM.

## 2 Methods

### 2.1 Ethics statement

As all analyses in this meta-analysis were based on previously published studies, ethical approval was not necessary.

### 2.2 Search strategy and study selection

We searched for studies published until April 2018 that compared the clinical effectiveness of laminectomy with instrumented fusion and laminoplasty for the treatment of MCSM. The databases used include PubMed, Embase, the Cochrane library, CNKI (Chinese database), VIP (Chinese database) and WANFANG (Chinese database). The languages were restricted to Chinese or English and only published articles were included. The following search terms were used:

(1) cervical spondylotic myelopathy or CSM or ossification of posterior longitudinal ligament or OPLL;
(2) laminoplasty;
(3) laminectomy or fusion or instrumentation; (1) and (2) and (3) in combination.

Reference lists of all included studies were scanned to identify additional potentially relevant studies. Two reviewers independently screened the titles and abstracts of identified papers, and full-text copies of all potentially relevant studies were obtained.

### 2.3 Inclusion criteria

Studies were included if they met the following criteria:

(1) study design: randomized or nonrandomized controlled studies or cohort studies;
(2) study population: patients with MCSM;
(3) purpose of interventions: to compare the differences in clinical outcomes between laminectomy with instrumented fusion and laminoplasty; and
(4) outcome measurements: with at least 1 desirable outcome.

Studies that did not meet the above criteria were excluded from selection.

### 2.4 Quality assessment of included studies

The Newcastle–Ottawa quality assessment scale (NOS) was used to evaluate the quality of the included studies.

### 2.5 Data extraction

The following information was extracted from each study:

(1) basic characteristics, including publication year, study design, patient age, enrollment number and follow-up time;
(2) primary outcome, presented as preoperative and postoperative Japanese Orthopedic Association (JOA) scores, preoperative and postoperative VAS, preoperative and postoperative ROM, preoperative and postoperative CCI, C5 nerve palsy rate and axial symptoms rate;
(3) secondary outcomes, including overall complication rate, operation time and blood loss.

### 2.6 Data analysis

We performed all meta-analyses with the Review Manager software (RevMan Version 5.3, The Nordic Cochrane Center, The Cochrane Collaboration, Copenhagen, Denmark). Heterogeneity was tested using the chi-square test and quantified by calculating the I^2^ statistic, for which *P* <.1 and I^2^ >50% was considered to be statistically significant. For the pooled effects, the standardized mean difference (SMD) was calculated for continuous variables and the risk ratio (RR) was calculated for dichotomous variables. Continuous variables are presented as SMD and 95% confidence intervals (CI), whereas dichotomous variables are presented as RR and 95% CI. Random-effects or fixed-effects models were used depending on the heterogeneity of the studies included.

## 3 Results

### 3.1 Search results and quality assessment

**Figure 1:**
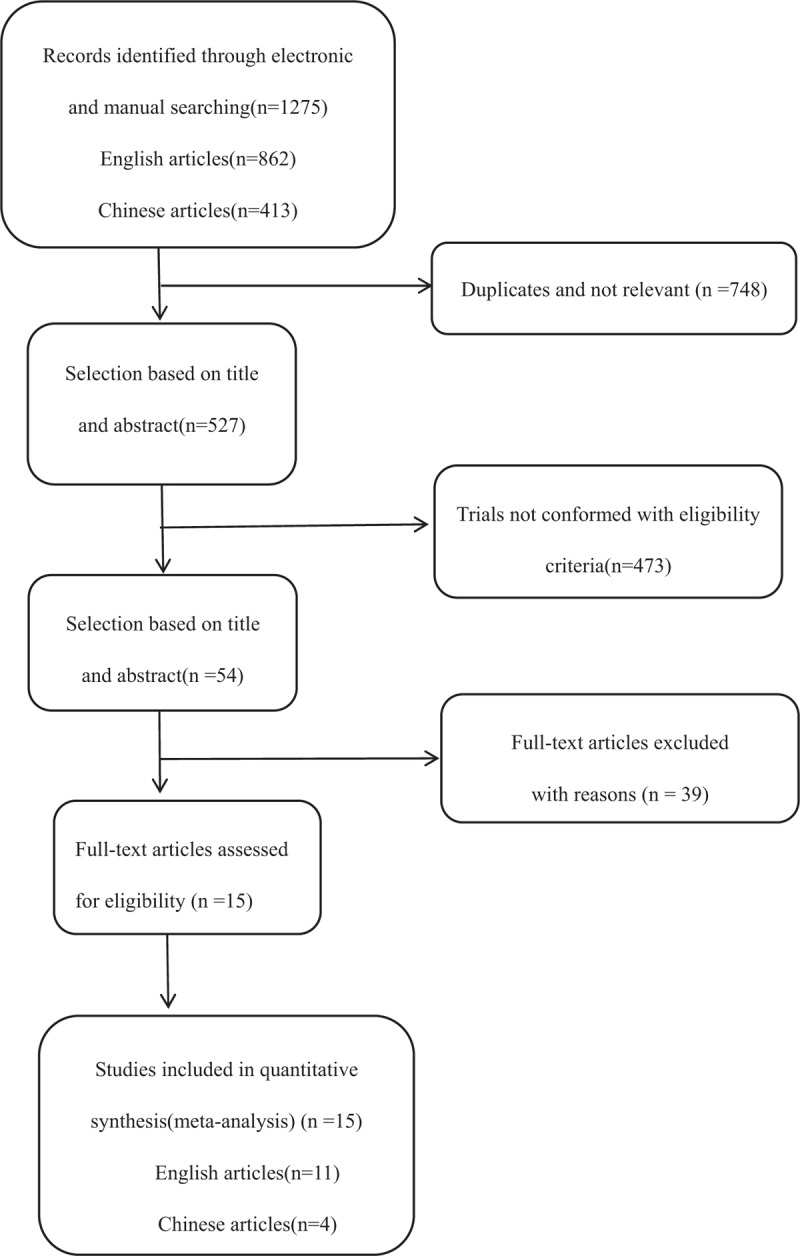
The flow chart showing the article selection process.

The process of identifying relevant studies is summarized in Figure 1. From the selected databases, 1275 references were obtained. By screening the titles and abstracts, 1221 references were excluded because they were duplicates, irrelevant studies, case reports, not comparative studies and reviews. The remaining 54 reports underwent a detailed and comprehensive evaluation. Eight systematic reviews or meta-analyses were not eligible because of a lack of primary data. Twelve studies were excluded because of comparisons with anterior surgical approaches. Seven studies were excluded because the laminectomy patients did not receive an instrumented fusion. Eight studies were excluded because patients underwent only laminoplasty or laminectomy. Four studies were excluded because they did not provide available data related to MCSM patients. Finally, 15 studies^[6–20]^ were included in this meta-analysis. Eleven studies^[6–16]^ were published in English, and the other 4 studies^[17–20]^ were published in Chinese. Table 1 and Table 2 summarize the assessment of baseline characteristics and quality of included studies, respectively. The NOS was used to assess the quality of each study. All studies scored 7 to 8 points, so the quality of each study was relatively high.

**Table 1:**
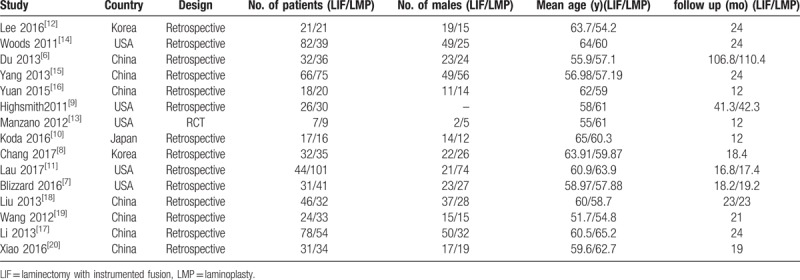
Characteristics of included studies.

**Table 2:**
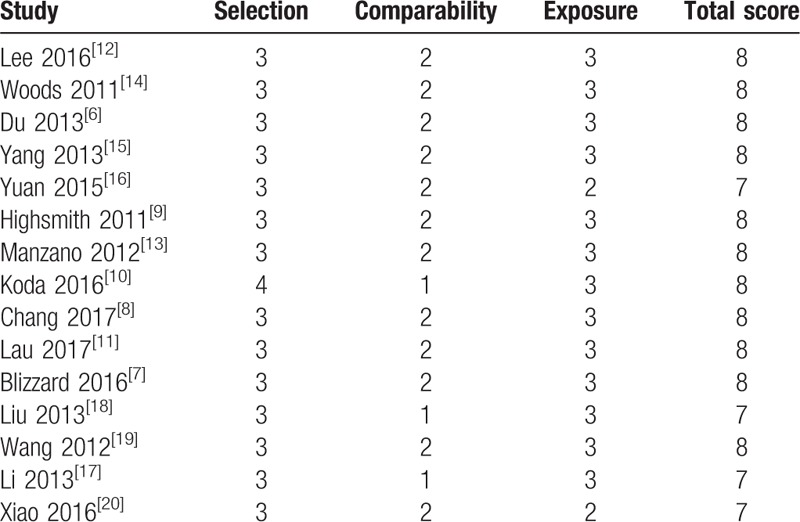
Quality assessment of included studies according to Newcastle–Ottawa scale (NOS).

### 3.2 Clinical evaluation

#### 3.2.1 Preoperative JOA scores

**Figure 2:**
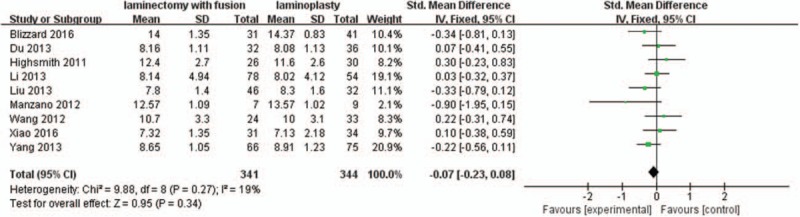
Forest plot of preoperative JOA scores between the LIF group and the LMP group. JOA = Japanese Orthopedic Association, LIF = laminectomy with instrumented fusion, LMP = laminoplasty.

Nine studies with a total of 685 patients (341 in the LIF group and 344 in the LMP group) provided preoperative JOA scores. The research exhibited no statistically significant heterogeneity (*P* = .27, I^2^ = 19%); therefore, a fixed effect model was used as the pooling method, and SMD was applied to analyze the overall effect. Preoperative JOA scores were similar between the 2 groups [SMD = −0.07, 95% CI: −0.23, 0.08; *P* = .34; Fig. 2].

#### 3.2.2 Postoperative JOA scores

**Figure 3:**
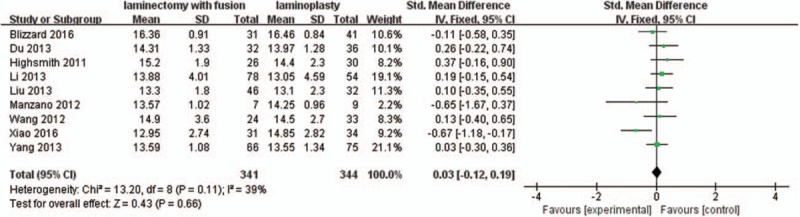
Forest plot of postoperative JOA scores between the LIF group and the LMP group. JOA = Japanese Orthopedic Association, LIF = laminectomy with instrumented fusion, LMP = laminoplasty.

Nine studies with a total of 685 patients (341 in the LIF group and 344 in the LMP group) provided postoperative JOA scores. The research exhibited no statistically significant heterogeneity (*P* = .11, I^2^ = 39%); thus, a fixed effect model was used as the pooling method, and SMD was applied to analyze the overall effect. Postoperative JOA scores were similar between the 2 groups [SMD = 0.03, 95% CI: −0.12, 0.19; *P* = .66; Fig. 3].

#### 3.2.3 Preoperative VAS scores

**Figure 4:**
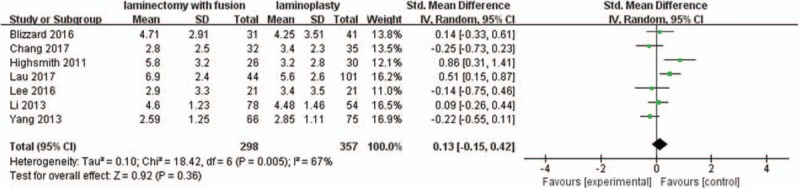
Forest plot of preoperative VAS scores between the LIF group and the LMP group. LIF = laminectomy with instrumented fusion, LMP = laminoplasty, VAS = visual analog scale.

Seven studies with a total of 655 patients (298 in the LIF group and 357 in the LMP group) provided preoperative VAS scores. The research exhibited statistically significant heterogeneity (*P* = .005, I^2^ = 67%); therefore, a random effect model was used as the pooling method, and SMD was applied to analyze the overall effect. Preoperative VAS scores were similar between the 2 groups [SMD = 0.13, 95% CI: −0.15, 0.42; *P* = .36; Fig. 4].

#### 3.2.4 Postoperative VAS scores

**Figure 5:**
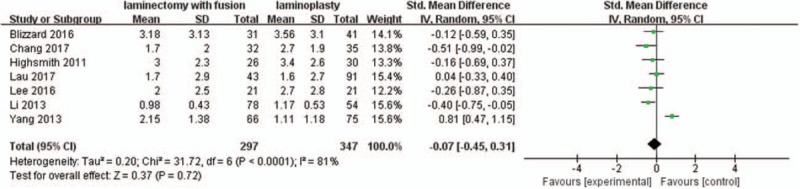
Forest plot of postoperative VAS scores between the LIF group and the LMP group. LIF = laminectomy with instrumented fusion, LMP = laminoplasty, VAS = visual analog scale.

Seven studies with a total of 644 patients (297 in the LIF group and 347 in the LMP group) provided postoperative VAS scores. The research exhibited statistically significant heterogeneity (*P* <.0001, I^2^ = 81%); therefore, a random effect model was used as the pooling method, and SMD was applied to analyze the overall effect. Postoperative VAS scores were similar between the 2 groups [SMD = −0.07, 95% CI: −0.45, 0.31; *P* = .72; Fig. 5].

#### 3.2.5 Preoperative CCI

**Figure 6:**
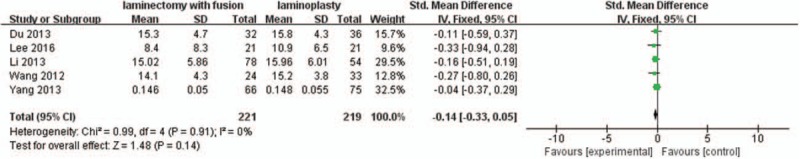
Forest plot of preoperative CCI between the LIF group and the LMP group. CCI = cervical curvature index, LIF = laminectomy with instrumented fusion, LMP = laminoplasty.

Five studies with a total of 440 patients (221 in the LIF group and 219 in the LMP group) provided preoperative CCI. The research exhibited no statistically significant heterogeneity (*P* = .91, I^2^ = 0%); therefore, a fixed effect model was used as the pooling method, and SMD was applied to analyze the overall effect. Preoperative CCI was similar between the 2 groups [SMD = −0.14, 95% CI: −0.33, 0.05; *P* = .14; Fig. 6].

#### 3.2.6 Postoperative CCI

**Figure 7:**
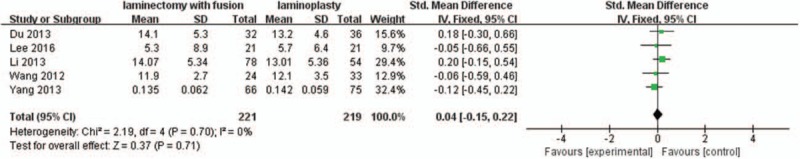
Forest plot of postoperative CCI between the LIF group and the LMP group. CCI = cervical curvature index, LIF = laminectomy with instrumented fusion, LMP = laminoplasty.

Five studies with a total of 440 patients (221 in the LIF group and 219 in the LMP group) provided postoperative CCI. The research exhibited no statistically significant heterogeneity (*P* = .70, I^2^ = 0%); therefore, fixed effect model was used as the pooling method, and SMD was applied to analyze the overall effect. Postoperative CCI was similar between the 2 groups [SMD = 0.04, 95% CI: −0.15, 0.22; *P* = .71; Fig. 7].

#### 3.2.7 Preoperative ROM

**Figure 8:**
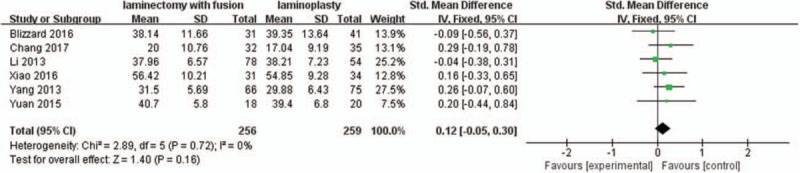
Forest plot of preoperative ROM between the LIF group and the LMP group. LIF = laminectomy with instrumented fusion, LMP = laminoplasty, ROM = range of motion.

Six studies with a total of 515 patients (256 in the LIF group and 259 in the LMP group) provided preoperative ROM. The research exhibited statistically significant heterogeneity (*P* = .72, I^2^ = 0%); therefore a fixed effect model was used as the pooling method, and SMD was applied to analyze the overall effect. Preoperative ROM was similar between the 2 groups [SMD = 0.12, 95% CI: −0.05, 0.30; *P* = .16; Fig. 8].

#### 3.2.8 Postoperative ROM

**Figure 9:**
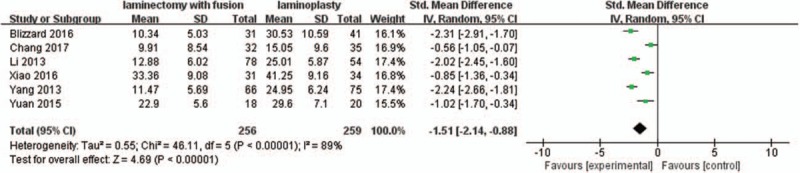
Forest plot of postoperative ROM between the LIF group and the LMP group. LIF = laminectomy with instrumented fusion, LMP = laminoplasty, ROM = range of motion.

Six studies with a total of 515 patients (256 in the LIF group and 259 in the LMP group) provided postoperative ROM. The research exhibited statistically significant heterogeneity (*P* <.00001, I^2^ = 89%); therefore, a random effect model was used as the pooling method, and SMD was applied to analyze the overall effect. The postoperative ROM was significantly greater in the LMP group compared with LIF group [SMD = −1.51, 95% CI: −2.14, −0.88; *P* <.00001; Fig. 9].

#### 3.2.9 C5 nerve palsy rate

**Figure 10:**
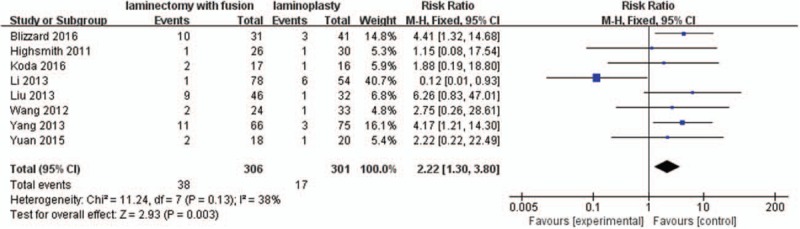
Forest plot of C5 nerve palsy rate between the LIF group and the LMP group. LIF = laminectomy with instrumented fusion, LMP = laminoplasty.

Eight studies with a total of 607 patients (306 in the LIF group and 301 in the LMP group) provided the C5 nerve palsy rate. The research exhibited no statistically significant heterogeneity (*P* = .13, I^2^ = 38%); therefore, a fixed effect model was used as the pooling method, and RR was applied to analyze the overall effect. The C5 nerve palsy rate was significantly higher in the LIF group compared with the LMP group [RR = 2.22, 95% CI: 1.30, 3.80; *P* = .003; Fig. 10].

#### 3.2.10 Axial symptoms rate

**Figure 11:**
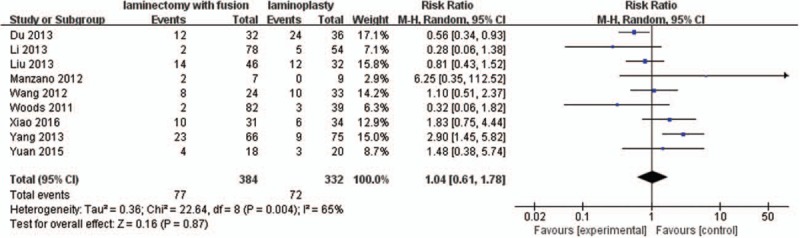
Forest plot of axial symptoms rate between the LIF group and the LMP group. LIF = laminectomy with instrumented fusion, LMP = laminoplasty.

Nine studies with a total of 716 patients (384 in the LIF group and 332 in the LMP group) provided the axial symptoms rate. The research exhibited statistically significant heterogeneity (*P* = .004, I^2^ = 65%); therefore, random effect model was used as the pooling method, and RR was applied to analyze the overall effect. The axial symptoms rate was similar between the 2 groups [RR = 1.04, 95% CI: 0.61, 1.78; *P* = .87; Fig. 11].

#### 3.2.11 Overall complication rate

**Figure 12:**
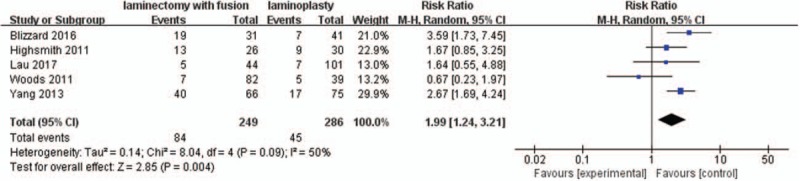
Forest plot of overall complication rate between the LIF group and the LMP group. LIF = laminectomy with instrumented fusion, LMP = laminoplasty.

Five studies with a total of 535 patients (249 in the LIF group and 286 in the LMP group) provided the overall complication rate. The research exhibited statistically significant heterogeneity (*P* = .09, I^2^ = 50%); therefore, a random effect model was used as the pooling method, and RR was applied to analyze the overall effect. The overall complication rate was significantly higher in the LIF group compared with the LMP group [RR = 1.99, 95% CI: 1.24, 3.21; *P* = .004; Fig. 12].

#### 3.2.12 Operation time

**Figure 13:**
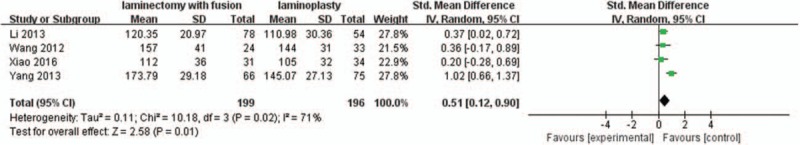
Forest plot of operation time between the LIF group and the LMP group. LIF = laminectomy with instrumented fusion, LMP = laminoplasty.

Four studies with a total of 395 patients (199 in the LIF group and 196 in the LMP group) provided the operation time. The research exhibited statistically significant heterogeneity (*P* = .02, I^2^ = 71%); therefore, a random effect model was used as the pooling method, and SMD was applied to analyze the overall effect. The operation time was significantly shorter in the LMP group compared with the LIF group [SMD = 0.51, 95% CI: 0.12, 0.90; *P* = .01; Fig. 13].

#### 3.2.13 Blood loss

**Figure 14:**
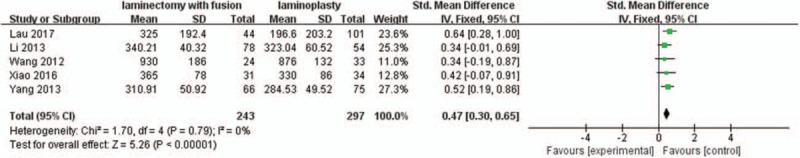
Forest plot of blood loss between the LIF group and the LMP group. LIF = laminectomy with instrumented fusion, LMP = laminoplasty.

Five studies with a total of 540 patients (243 in the LIF group and 297 in the LMP group) provided blood loss. The research exhibited no statistically significant heterogeneity (*P* = .79, I^2^ = 0%); therefore, a fixed effect model was used as the pooling method, and SMD was applied to analyze the overall effect. The blood loss was significantly lower in the LMP group compared with the LIF group [SMD = 0.47, 95% CI: 0.30, 0.65; *P* <.00001; Fig. 14].

#### 3.2.14 Sensitivity analysis

Sensitivity analysis was performed to confirm the stability of this meta-analysis by sequentially omitting individual eligible studies. The pooled results were not significantly changed after each study was excluded, which showed the stability of the results.

#### 3.2.15 Publication bias

**Figure 15:**
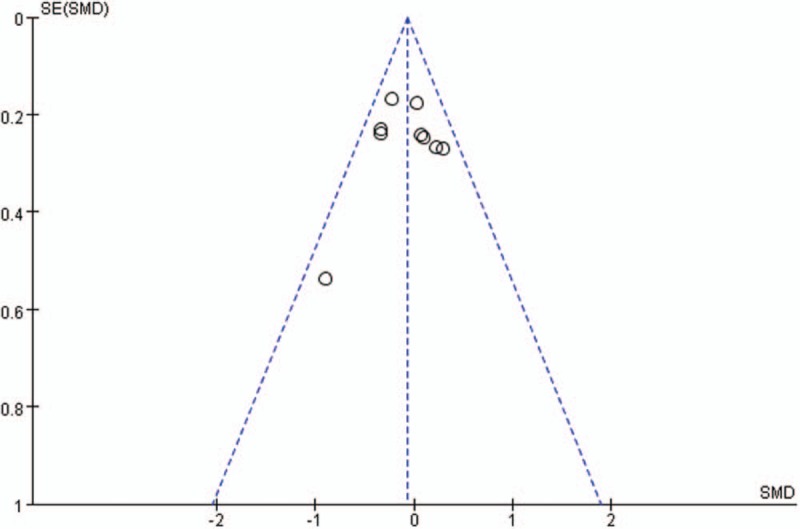
Funnel plots for preoperative JOA scores. JOA = Japanese Orthopedic Association.

**Figure 16:**
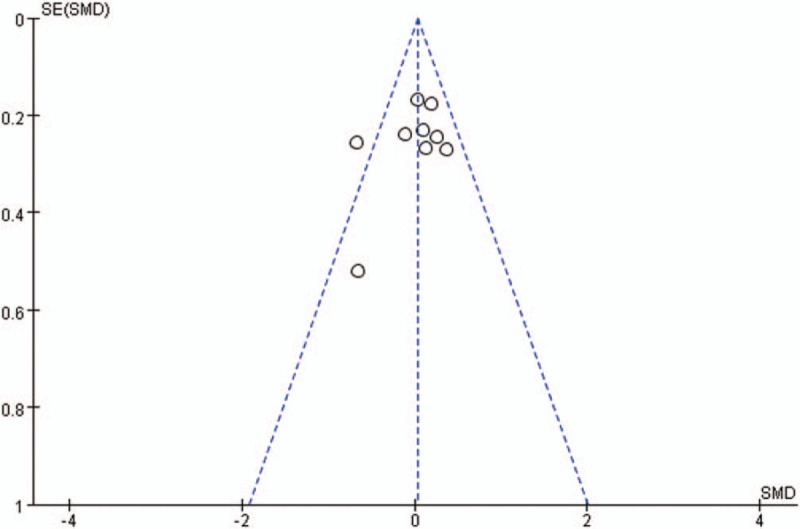
Funnel plots for postoperative JOA scores. JOA = Japanese Orthopedic Association.

**Figure 17:**
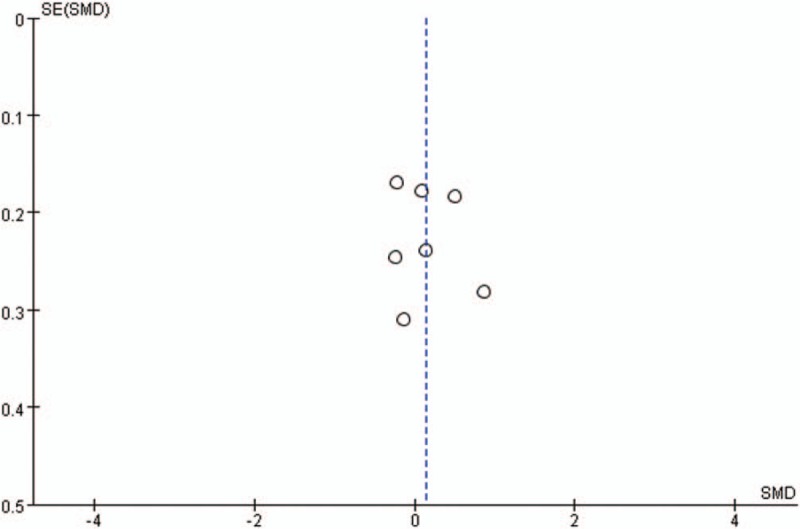
Funnel plots for preoperative VAS scores. VAS = visual analog scale.

**Figure 18:**
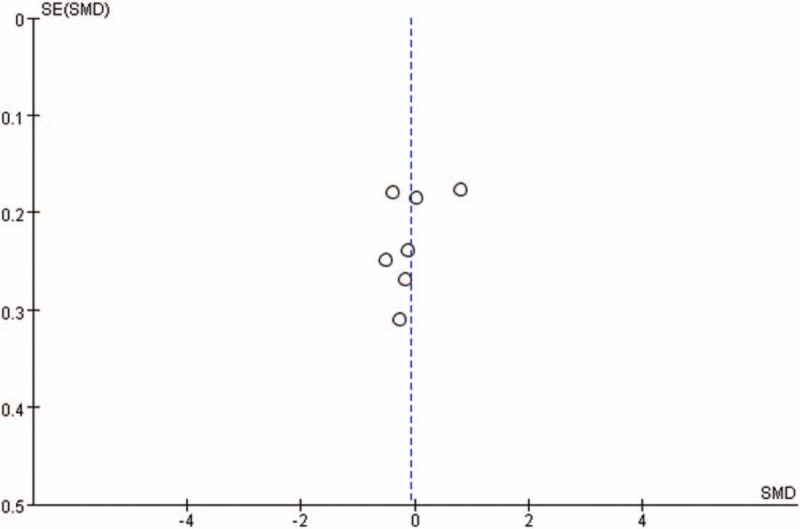
Funnel plots for postoperative VAS scores. VAS = visual analog scale.

Publication bias for included studies was assessed by funnel plots (Figs. 15–18). Funnel plots appeared nearly symmetrical for preoperative JOA scores, postoperative JOA scores, preoperative VAS scores, and postoperative VAS scores, indicating no significant publication bias among the included studies.

## 4 Discussion

Surgical treatments for MCSM are complicated and challenging.^[7]^ Posterior approaches can provide satisfactory clinical outcomes in treating MCSM by indirect decompression of the spinal cord. Laminectomy with instrumented fusion and laminoplasty has been the most widely used posterior procedure. Previous systematic reviews comparing laminoplasty and laminectomy with instrumented fusion revealed a trend that laminoplasty was preferable with fewer total complications, including lower C5 nerve palsy and reduced surgical trauma, but could not determine which technique is superior.^[21–23]^ In addition, the relevant literature was only searched up to January 2016. Furthermore, they extracted limited data for quantitative analysis. Over the last 2 years, a few studies on posterior approaches for the treatment of MCSM have been published. Therefore, it remains necessary to verify the above conclusion based on the latest high-quality studies.

In this meta-analysis, from the literature search up to April 2018, we combined 15 studies that included a total of 555 patients in the laminectomy group and 576 patients in the laminoplasty group. Compared to laminectomy with instrumented fusion, laminoplasty showed better postoperative cervical ROM (SMD = 1.51) and fewer overall surgical complications (RR = 0.50), including decreased C5 palsy (RR = 0.45), shorter operation time (SMD = −0.51) and decreased blood loss (SMD = -0.47).

Lee et al^[21]^ published a meta-analysis of studies comparing laminoplasty with laminectomy for treating MCSM. In this article, the authors focused on the clinical and radiological outcomes between these 2 different methods. The authors suggest that both methods may obtain clinical improvement and lead to a similar loss of lordosis, but definitive conclusion could not be reached regarding which surgical approach is more effective for the treatment of MCSM. Phan et al^[23]^ also demonstrated that there is no clear advantage for either laminectomy with fusion or laminoplasty when treating patients with MCSM. However, a higher nerve palsy complication rate was found in laminectomy with fusion. Results of another study showed that both laminoplasty and laminectomy with fusion may achieve clinical improvement and a similar loss of lordosis. However, laminoplasty showed shorter operation time and lower C5 palsy rate.^[22]^ Our results are similar to those reported by others.

JOA scores and VAS are widely applied to assess the improvement of postoperative clinical outcomes. The Japanese Orthopaedic Association (JOA) score was developed by the JOA in 1975 and has become one of the most commonly used outcome measures to assess the functioning of patients with CSM. The visual analog scale (VAS) is commonly used to evaluate human psychology such as pain, and patients can rate the level of pain with a numerical scale. The pooled data showed that there was no statistically significant difference in preoperative JOA scores, preoperative VAS, postoperative JOA scores and postoperative VAS between the 2 groups, indicating that both of the 2 procedures can obtain sufficient spinal canal decompression and clinical improvement.

Cervical lordosis was measured on lateral X-ray by using the cervical curvature index (CCI) as described by Ishihara, and ROM was determined for C2-C7 by means of the Cobb method.^[15]^ The pooled data showed that there was no statistically significant difference in preoperative ROM, preoperative and postoperative CCI between the 2 groups. However, there was a statistically significant difference in postoperative ROM between the 2 groups, which indicated laminoplasty was superior to laminectomy in preserving cervical ROM. The reasons for this finding may be that multilevel rigid fixation and fusion procedures in laminectomy obviously restrict the range of movement of the cervical spine.

Axial symptoms include chronic neck and shoulder pain, stiffness, limitation of movement, and other symptoms following cervical surgery.^[2]^ C5 palsy could result in muscle weakness and numbness of the upper limbs after cervical decompression surgery.^[24]^ Axial symptoms and C5 palsy are considered the most important complications of cervical posterior procedures. The pooled data showed that there was no statistically significant difference in axial symptoms between the 2 groups. However, there was a statistically significant difference in the C5 palsy rate between the 2 groups, which indicated laminoplasty was superior to laminectomy with instrumented fusion in reducing the incidence of C5 palsy. After posterior decompression, the spinal cord drifts and the C5 nerve roots are tethered. In laminoplasty, the limited open-door angle of the lamina restricts the spinal cord drift to some extent. However, extensive spinal canal decompression in laminectomy with fusion worsens the tethering effect of the C5 nerve roots.^[24,25]^

Overall complication rate, operation time and blood loss are very important aspects for evaluating surgical trauma. The pooled data showed that there was a statistically significant difference in overall complication rate, operation time and blood loss between the 2 groups, which indicated that laminectomy with instrumented fusion, was associated with greater surgical trauma. For older patients with underlying diseases, laminoplasty may be more suitable.

We believe that the results of this meta-analysis are affected by several factors. First, only one of the included studies was a randomized controlled trial (RCT). Second, there was variability in choosing the indicators to evaluate clinical outcomes between the included studies, indicating a lack of standard outcome measurements. Third, the length of follow-up varied between studies, and this is important for surgical outcome evaluations. Finally, clinical heterogeneity might be caused by the various indications for operations.

## 5 Conclusion

Both laminectomy with instrumented fusion and laminoplasty are effective treatments for MCSM. Compared with laminectomy with instrumented fusion, laminoplasty appears to provide better clinical and radiographic outcomes with fewer surgical complications in treating MCSM. However, future well-designed, randomized controlled trials are still needed to further confirm our results.

## Author contributions

**Conceptualization**: Xiang Lin, Zengming Xiao.

**Data curation:** Xiang Lin, Jie Cai.

**Formal analysis:** Xiang Lin, Jie Cai, Zengming Xiao.

**Funding acquisition:** Zengming Xiao.

**Investigation:** Xiang Lin, Jie Cai, Chuan Qin.

**Methodology:** Xiang Lin, Jie Cai, Chuan Qin, Zengming Xiao.

**Project administration:** Xiang Lin, Jie Cai, Chuan Qin, Zengming Xiao.

**Resources:** Qinghua Yang, Zengming Xiao.

**Software:** Xiang Lin, Chuan Qin, Qinghua Yang.

**Supervision:** Zengming Xiao.

**Validation:** Xiang Lin, Qinghua Yang, Zengming Xiao.

**Visualization:** Xiang Lin, Qinghua Yang, Zengming Xiao.

**Writing – original draft:** Xiang Lin.

**Writing – review & editing:** Xiang Lin, Zengming Xiao.

## References

[1] Karadimas SK Gatzounis G Fehlings MG Pathobiology of cervical spondylotic myelopathy. Eur Spine J 2015;24:132–8. 24626958 10.1007/s00586-014-3264-4

[2] Rao RD Gourab K David KS Operative treatment of cervical spondylotic myelopathy. J Bone Joint Surg Am 2006;88:1619–40. 16818991 10.2106/JBJS.F.00014

[3] Xu L Sun H Li Z Anterior cervical discectomy and fusion versus posterior laminoplasty for multilevel cervical myelopathy: a meta-analysis. Int J Surg 2017;48:247–53. 28687344 10.1016/j.ijsu.2017.06.030

[4] Rhee JM Basra S Posterior surgery for cervical myelopathy: laminectomy, laminectomy with fusion, and laminoplasty. Asian Spine J 2008;2:114–26. 20404967 10.4184/asj.2008.2.2.114 PMC2852088

[5] Hirabayashi K Watanabe K Wakano K Expansive open-door laminoplasty for cervical spinal stenotic myelopathy. Spine (Phila Pa 1976) 1983;8:693–9. 6420895 10.1097/00007632-198310000-00003

[6] Du W Wang L Shen Y Long-term impacts of different posterior operations on curvature, neurological recovery and axial symptoms for multilevel cervical degenerative myelopathy. Eur Spine J 2013;22:1594–602. 23508336 10.1007/s00586-013-2741-5 PMC3698356

[7] Blizzard DJ Caputo AM Sheets CZ Laminoplasty versus laminectomy with fusion for the treatment of spondylotic cervical myelopathy: short-term follow-up. Eur Spine J 2017;26:85–93. 10.1007/s00586-016-4746-3 27554354

[8] Chang H Kim C Choi BW Selective laminectomy for cervical spondylotic myelopathy: a comparative analysis with laminoplasty technique. Arch Orthop Trauma Surg 2017;137:611–6. 28289891 10.1007/s00402-017-2670-6

[9] Highsmith JM Dhall SS Haid RJ Treatment of cervical stenotic myelopathy: a cost and outcome comparison of laminoplasty versus laminectomy and lateral mass fusion. J Neurosurg Spine 2011;14:619–25. 21388285 10.3171/2011.1.SPINE10206

[10] Koda M Mochizuki M Konishi H Comparison of clinical outcomes between laminoplasty, posterior decompression with instrumented fusion, and anterior decompression with fusion for K-line (-) cervical ossification of the posterior longitudinal ligament. Eur Spine J 2016;25:2294–301. 27072553 10.1007/s00586-016-4555-8

[11] Lau D Winkler EA Than KD Laminoplasty versus laminectomy with posterior spinal fusion for multilevel cervical spondylotic myelopathy: influence of cervical alignment on outcomes. J Neurosurg Spine 2017;27:508–17. 28862572 10.3171/2017.4.SPINE16831

[12] Lee CH Jahng TA Hyun SJ Expansive laminoplasty versus laminectomy alone versus laminectomy and fusion for cervical ossification of the posterior longitudinal ligament: is there a difference in the clinical outcome and sagittal alignment. Clin Spine Surg 2016;29:E9–15. 25075990 10.1097/BSD.0000000000000058

[13] Manzano GR Casella G Wang MY A prospective, randomized trial comparing expansile cervical laminoplasty and cervical laminectomy and fusion for multilevel cervical myelopathy. Neurosurgery 2012;70:264–77. 10.1227/NEU.0b013e3182305669 22251974

[14] Woods BI Hohl J Lee J Laminoplasty versus laminectomy and fusion for multilevel cervical spondylotic myelopathy. Clin Orthop Relat Res 2011;469:688–95. 21089002 10.1007/s11999-010-1653-5 PMC3032861

[15] Yang L Gu Y Shi J Modified plate-only open-door laminoplasty versus laminectomy and fusion for the treatment of cervical stenotic myelopathy. Orthopedics 2013;36:e79–87. 23276358 10.3928/01477447-20121217-23

[16] Yuan W Zhu Y Liu X Postoperative three-dimensional cervical range of motion and neurological outcomes in patients with cervical ossification of the posterior longitudinal ligament: cervical laminoplasty versus laminectomy with fusion. Clin Neurol Neurosurg 2015;134:17–23. 25929463 10.1016/j.clineuro.2015.04.004

[17] Li L Yan S Yu X Clinical mid-term effect of surgical posterior decompression methods on cervical curvature and intervertebral height. Orthop J Chin 2013;21:1929–36.

[18] Liu X Chen D Wang X The influence of K-line on two posterior approaches for patient with ossification of posterior longitudinal ligament. Chin J Spine Spinal Cord 2013;23:6–10.

[19] Wang H Ding W Shen Y Analysis of axial symptoms after indirect decompression for ossification of the posterior longitudinal ligament of the cervical spine. Chin J Surg 2012;50:601–6. 22943989

[20] Xiao J Tang G Comparison of the efficacy between posterior single unilateral open-door laminoplasty using mini-plate fixation and laminectomy with lateral mass screw fixation in the treatment of multi-segmental cervical spondylotic myelopathy. Orthopaedics 2016;7:168–71.

[21] Lee CH Lee J Kang JD Laminoplasty versus laminectomy and fusion for multilevel cervical myelopathy: a meta-analysis of clinical and radiological outcomes. J Neurosurg Spine 2015;22:589–95. 25815808 10.3171/2014.10.SPINE1498

[22] Liu FY Yang SD Huo LS Laminoplasty versus laminectomy and fusion for multilevel cervical compressive myelopathy: a meta-analysis. Medicine (Baltimore) 2016;95:e3588. 27281067 10.1097/MD.0000000000003588 PMC4907645

[23] Phan K Scherman DB Xu J Laminectomy and fusion vs laminoplasty for multi-level cervical myelopathy: a systematic review and meta-analysis. Eur Spine J 2017;26:94–103. 27342611 10.1007/s00586-016-4671-5

[24] Shou F Li Z Wang H Prevalence of C5 nerve root palsy after cervical decompressive surgery: a meta-analysis. Eur Spine J 2015;24:2724–34. 26281981 10.1007/s00586-015-4186-5

[25] Wang T Wang H Liu S Incidence of C5 nerve root palsy after cervical surgery: a meta-analysis for last decade. Medicine (Baltimore) 2017;96:e8560. 29137073 10.1097/MD.0000000000008560 PMC5690766

